# Flavonoids with α-glucosidase inhibitory activities and their contents in the leaves of *Morus atropurpurea*

**DOI:** 10.1186/1749-8546-8-19

**Published:** 2013-10-14

**Authors:** Hin-Chu Hong, Song-Lin Li, Xiao-Qi Zhang, Wen-Cai Ye, Qing-Wen Zhang

**Affiliations:** 1 State Key Laboratory of Quality Research in Chinese Medicine, Institute of Chinese Medical Sciences, University of Macau, Macao, China; 2 Department of Pharmaceutical Analysis and Metabolomics, Jiangsu Province Academy of Traditional Chinese Medicine, Nanjing, 210028, China; 3 Institute of Traditional Chinese Medicine and Natural Products, Jinan University, Guangzhou, 510632, China

## Abstract

**Background:**

This study aims to isolate the α-glucosidase inhibitory compounds from mulberry leaves (*Morus atropurpurea* Roxb., Moraceae) and to develop an analytical method for quantification of the compounds.

**Methods:**

Four flavonoids, rutin (**1**), isoquercetin (**2**), kaempferol-3-*O*-rutinoside (**3**) and astragalin (**4**), were isolated by column chromatography from mulberry leaf water extracts (MWE). The α-glucosidase inhibitory activities of MWE and the four isolated compounds were evaluated by a microplate-based *in vitro* assay. The content of the isolated flavonoids in *M*. *atropurpurea* leaves purchased from different local herbal stores or collected in different locations was determined by high performance liquid chromatography.

**Results:**

The four flavonoids (**1**–**4**) showed α-glucosidase inhibitory activities, with rutin (**1**) and astragalin (**4**) showing high α-glucosidase inhibitory activities (IC_50_ values of 13.19 ± 1.10 and 15.82 ± 1.11 μM, respectively). The total contents of the four flavonoids were different among eight samples examined, ranging from 4.34 mg/g to 0.53 mg/g.

**Conclusions:**

The four flavonoids in *M*. *atropurpurea* leaves could inhibit α-glucosidase activity.

## Background

Postprandial hyperglycemia is one of the earliest abnormalities of glucose homoeostasis [1]. The production and absorption of glucose can be decreased through the inhibition of a carbohydrate hydrolyzing enzyme, α-glucosidase [2]. Water extracts from some species of mulberry leaves (Moraceae) show potent antihyperglycemic activities *via* α-glucosidase inhibition [3-5]. It was subsequently found that this α-glucosidase inhibition was attributed to the actions of iminosugars such as 1-deoxynojirimycin (1-DNJ) and N-methyl-1-deoxynojirimycin (N-methyl-1-DNJ) [6,7]. Flavonoids, which are widely distributed in the plant kingdom, have also been shown to decrease blood glucose levels [2,8].

This study aims to isolate the α-glucosidase inhibitory compounds from mulberry leaves (*Morus atropurpurea* Roxb., Moraceae) and to develop an analytical method for quantification of the compounds. The α-glucosidase inhibitory activity of *M*. *atropurpurea* leaf water extracts (MWE) was evaluated. Four flavonoids, rutin (**1**), isoquercetin (**2**), kaempferol-3-*O*-rutinoside (**3**), and astragalin (**4**), were isolated from *M*. *atropurpurea* Roxb. leaves purchased from different local herbal stores or collected in different locations. The content of individual flavonoids in *M*. *atropurpurea* leaves was determined by high performance liquid chromatography (HPLC).

## Methods

### Materials and chemicals

Acetonitrile (HPLC grade) and acetic acid (HPLC grade) were purchased from Merck (Darmstadt, Germany), and methanol (AR grade) was purchased from Kaitong (Tianjin, China). Sodium carbonate was purchased from BDH Laboratory Supplies (Poole, Dorset, UK). α-Glucosidase type 1 (EC 3.2.1.200 from *Saccharomyces cerevisias*) and 4-nitrophenyl α-D-glucopyranoside (pNPG) were purchased from Sigma-Aldrich (St. Louis, MO, USA). Ultra-pure water was purified using a Millipore Milli Q-Plus system (Billerica, MA, USA). Reference compounds of rutin (1), isoquercetin (2), kaempferol-3-*O*-rutinoside (3), astragalin (4) were isolated from *M*. *atropurpurea*. Silica gel (200–300 mesh) was purchased from Qingdao Haiyang Chemical Co. Ltd. (Qingdao, China). Macroporous absorption resin (D101) was purchased from Haiguang Chemical Industrial Co. (Tianjin, China). Sephadex LH-20 was purchased from GE Healthcare Bio-Sciences AB (Uppsala, Sweden), and the Millex® nylon membrane syringe filter was purchased from Millipore (Billerica, MA, USA).

Samples of *M*. *atropurpurea* (10 kg of leaves of the sample for separation and 500 g of each of the samples for analysis) were purchased from local herbal stores or collected arbitrarily in Guangdong Province, China. The botanical origin of the raw materials was authenticated by Professor Guang-Xiong Zhou at the College of Pharmacy, Jinan University, Guangzhou, China by morphological identification [9,10]. Voucher specimens (ICMS20110421) were deposited in the Institute of Chinese Medical Sciences, University of Macau, Macau SAR, China.

### Apparatus

Electrospray injection mass spectrometry (ESI-MS) was performed on an Agilent 1100 LC/MSD Trap Mass Spectrometer (Agilent, USA), and ^1^H ^13^C NMR spectra were obtained using a 300 MHz NMR spectrometer (Bruker, Germany). HPLC analyses were performed using an Agilent 1200 HPLC (Agilent, USA) system. Extraction was carried out using the Syncore Polyvap Analyst and Reactor (BUCHI, Switzerland). The absorbance readings in enzymatic assays were recorded by a SpectraMax® M5 multi-mode microplate reader (Molecular Devices, USA).

### Isolation

Nine kg of air-dried *M*. *atropurpurea* leaves were extracted with boiling water (3 × 20 L, 1 h each). The extract was filtered and subjected to a D101 macroporous adsorption resin column, then eluted with H_2_O, 30% EtOH, 40% EtOH, 60% EtOH and 100% EtOH (v/v, H_2_O-EtOH) sequentially. The 40% EtOH fraction was concentrated to dryness and then suspended in H_2_O. The water suspension was partitioned with EtOAc (v/v, H_2_O-EtOAc = 1:1) 3 times to obtain EtOAc fractions, while the remaining H_2_O fraction was partitioned with BuOH (v/v, H_2_O-BuOH = 2:1) three more times to obtain BuOH fractions. The EtOAc fraction was concentrated to dryness and subjected to silica gel chromatography column, using CHCl_3_-MeOH-H_2_O (from CHCl_3_-MeOH-H_2_O = 20:1:0 to CHCl_3_-MeOH-H_2_O = 70:30:5) as eluent to afford 40 fractions (E1–E40). Fractions E25–E29 were further separated by a Sephadex LH-20 column eluted with methanol to afford flavonoid **4** (20 mg). Fractions E30–E40 were further separated by a Sephadex LH-20 column eluted with methanol to afford 20 fractions (Ea1–Ea20). Fractions Ea1–Ea20 were further purified by preparative HPLC (C18, column flow rate = 10 mL/min 45% MeOH 30 min) to afford flavonoid **2** (15 mg). The BuOH fractions were concentrated to dryness and subjected to a silica gel column using CHCl_3_-MeOH as eluent to afford 24 fractions (B1–B24). Fractions B17–B24 were further purified by a Sephadex LH-20 column eluted with methanol to afford flavonoid **3** (30 mg). Fractions B8–16 were further purified by a Sephadex LH-20 column eluted with methanol to afford flavonoid **1** (15 mg).

### α-glucosidase assay

The yeast α-glucosidase inhibitory activities of the four flavonoids were determined by a microplate-based method [11] with a slight modification. In the sample group, a total of 80 μL of reaction mixture contained 20 μL each of 100 mM phosphate buffer (pH 6.8), 2.5 mM pNPG in the buffer, experimental drug and 2.4 U/mL α-glucosidase. After incubation of the 96-well plates at 37°C for 30 min, 80 μL of 0.2 mol/L sodium carbonate solution was added to each well to stop the reaction. The 4-nitrophenol absorption was measured at 405 nm. In the control group, the composition of the reaction mixture was the same as in sample group except that solvent was used instead of the experimental drug. In the sample and control blank groups, the composition was same as in the sample group except that α-glucosidase was used instead with buffer. The inhibitory activity was calculated using the following formula:

$$\mathrm{Inhibitory}\;\mathrm{activity}\left(\%\right)=1–\left(A_{S}–A_{\mathrm{SB}}\right)/\left(A_{C}–A_{\mathrm{CB}}\right)\times 100$$

where A_S_, A_SB_, A_C_, and A_CB_ are the absorbances of sample, sample blank, control, and control blank, respectively.

### Sample preparation

The leaves of *M*. *atropurpurea* were refluxed on a Syncore Polyvap Analyst Reactor under optimized conditions. Dried powder of *M*. *atropurpurea* leaves (0.4 g) was refluxed (100°C) in 20 mL of methanol for 1 h. After extraction, solvent was added to the extraction vessel until the final weight equal to the starting weight to counter solvent loss. The extract was thoroughly mixed on a vortex mixer, and filtered through a 0.45 μm syringe filter prior to HPLC injection.

### Optimization of the sample preparation conditions

A single factor test was performed by changing one factor, fixing other factors to optimize the sample preparation conditions for the analysis. Five factors, including temperature, extraction time, particle size of raw materials (RM), raw material to solvent ratio (RMTSR), and extraction cycle were examined. For RMTSR, the RMTSR was changed from 1:20 (g:mL) to 1:150 (g:mL), while other factors were set as follows: extraction time 1 h, extraction temperature 100°C, and particle size between 124 and 150 μm. The temperature was changed from 70 to 100°C, while other factors were set as follows: extraction time 1 h, RMTSR of 1:100, and particle size between 124 and 150 μm. The extraction time was changed from 30 min to 2 h, while other factors were set as follows: RMTSR of 1:100, extraction temperature 100°C, and particle size between 124 and 150 μm. The particle size of RM was changed from less than 420 μm to between 124 and 150 μm, while other factors were set as follows: extraction time 1 h, extraction temperature 100°C, and RMTSR of 1:100. For optimization of the extraction cycle, the RM was extracted in 1–3 cycles, while other factors were set as follows: extraction time 1 h, extraction temperature 100°C, RMTSR of 1:100 and particle size between 124 and 150 μm. The yield of the four analytes was the criterion for the optimization procedures.

### Chromatographic conditions

All experiments were conducted with an Agilent 1200 HPLC system. The mobile phase consisted of water with 0.5% acetic acid (A) and acetonitrile (B). The gradient programs were as follows: 0–25 min, 18–19% B; 25–30 min, 100% B. The chromatographic separation was using a Cosmosil 5 C_18_-MS-II (4.6 × 250 mm, 5 μm) column with a flow rate of 1 mL/min. The column temperature was maintained at 30°C. All analytes were monitored at 350 nm.

### Calibration curves

Stock standard solutions of four flavonoids were prepared in methanol and diluted to different concentrations to build calibration curves. The calibration curves were constructed by plotting the peak areas versus the concentrations of each analyte.

### Precision test

Intra-day and inter-day variations were used to determine the precision of the developed method. The intra-day precision or inter-day precision was determined by analyzing replicated samples (MA06) within 1 day or within 3 consecutive days, respectively.

### Stability test

Sample MA06 was analyzed using the developed method to verify the stability of the samples. The stability test was carried out by analyzing the sample at 0, 2, 4, 8, 12, 24, and 48 h, and the relative standard deviations (RSDs) of peak areas at different times were calculated.

### Accuracy test

The accuracy of the developed method was evaluated by spike recovery. Flavonoids **1**, **2**, **3**, and **4** were added into 0.2 g of sample MA06. Then, the mixtures were extracted and analyzed. The spiked recovery was calculated as follows:

$$\begin{array}{l} \mathrm{Recovery}\;\left(\%\right)=\left(\mathrm{amount}\;\mathrm{found}-\mathrm{amount}\;\mathrm{original}\right)\\ \;÷\mathrm{amount}\;\mathrm{spiked}\times 100\% \end{array}$$

### Statistical analysis

The results are reported as the means ± standard derivations (SD) of at least three measurements. Significant variables were assessed using the *t* test, subjecting results to a simple linear regression in Excel (Microsoft, Redmond, WA, USA). Results with *P* values less than 0.05 were considered to be significant.

## Results and discussion

### Structural determination

**Figure 1:**
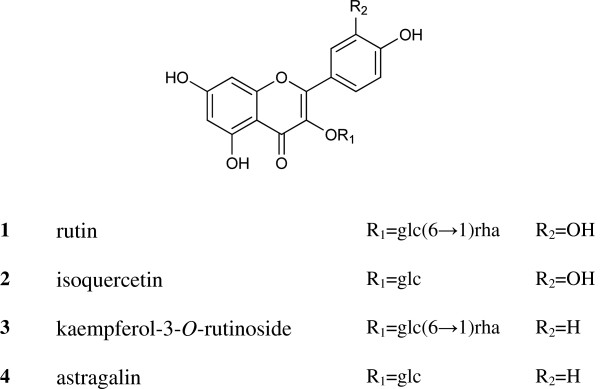
**Structures of the four flavonoids (1–4) extracted from *M*. *atropurpurea*.**

Four flavonoids isolated from the *M*. *atropurpurea* leaves were identified as rutin (**1**), isoquercetin (**2**), kaempferol-3-O-rutinoside (**3**), and astragalin (**4**) (shown in Figure 1) by comparing the MS and NMR data with the literature [10]. The ^1^H NMR spectra revealed that their purities were all above 98%, which was also confirmed by HPLC.

### α-glucosidase assay

The α-glucosidase assay used acarbose as a positive control with a 50% inhibitory concentration (IC_50_) of 780.2 ± 1.04 μM. As shown in Table 1, all of the tested flavonoids inhibited yeast α-glucosidase in a dose-dependent manner. The dose-dependent manner was determined by the dose-inhibition relationship experiment. A series sample concentration was tested to determine the IC_50_. The IC_50_ for flavonoids **1**–**4** were determined to be 13.19 ± 1.10, 116.7 ± 1.17, 365.4 ± 1.05 and 15.82 ± 1.11 μM, respectively. The IC_50_ for MWE was 27.05 ± 1.04 μg/mL.

**Table 1:** Inhibitory activities of the four flavonoids against α-glucosidase

**Samples**	**IC**_**50**_**, μM**	**IC**_**50**_**, μg/mL**
**Acarbose**	780.2 ± 1.04	503.7 ± 0.67
**1**	13.19 ± 1.10	8.05 ± 0.67
**2**	116.7 ± 1.17	54.19 ± 0.54
**3**	365.4 ± 1.05	217.24 ± 0.62
**4**	15.82 ± 1.11	7.09 ± 0.28
**WME**	-	27.05 ± 0.50

Data are presented as means ± SD of three measurements (n = 3).

### Optimization of the sample preparation conditions

**Figure 2:**
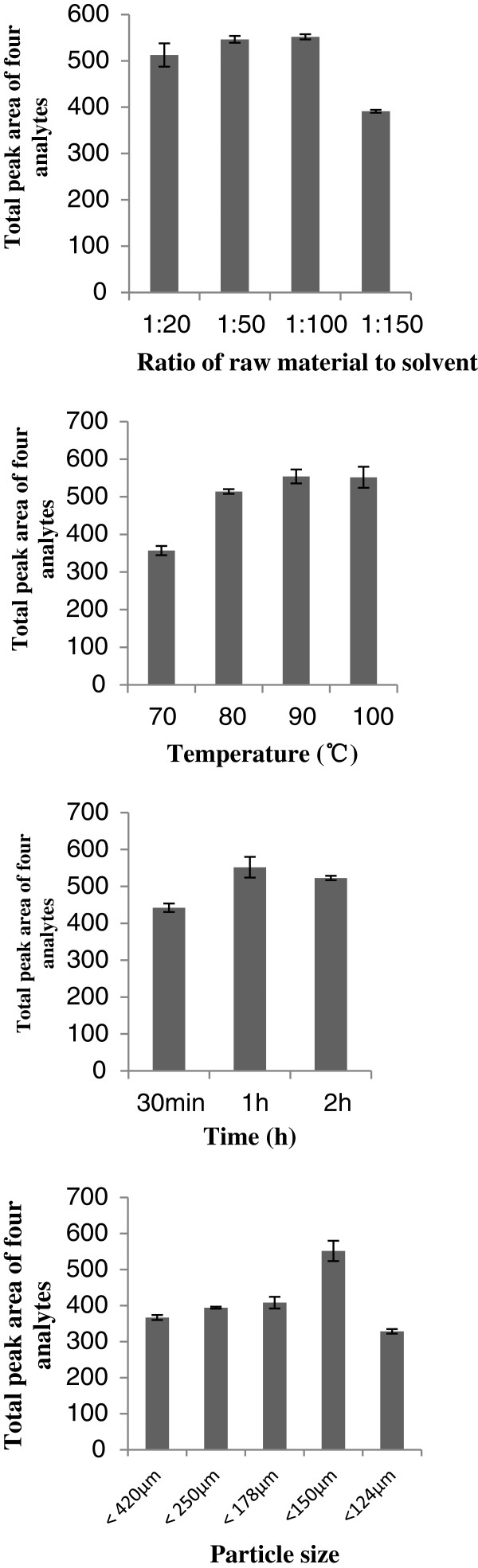
Optimization of sample preparation conditions.

As shown in Figure 2, the effects of the raw material to solvent ratio (RMTSR), temperature, extraction time, and extraction cycle were investigated. The yield of four analytes increased with RMTSR until a ratio of 1:100. The maximum extraction yield was obtained by the RMTSR of 1:100; nevertheless, there was no significant difference between 1:50 and 1:100. The yield of the four analytes increased with temperature from 70 to 90°C. The maximum extraction yield was obtained at 100°C; nevertheless, there was no significant difference between 90 and 100°C. The maximum extraction yield was obtained at 1 h, suggesting that 1 h is long enough for complete extraction of the analytes. The yield of the four analytes increased with decreasing particle sizes until the sizes were between 124 and 150 μm. The maximum extraction yield was obtained with particle sizes between 124 and 150 μm. The extraction rate of the second extraction was less than 5% of that of the first extraction, indicating that one extraction cycle was enough for complete extraction.

The results of the single factor test indicated that the extraction conditions could be optimized as follows: RMTSR, 1:100; temperature, 100°C; extraction time, 1 h; particle size of RM, between 124 and 150 μm; extraction cycles, 1.

### HPLC method development

**Figure 3:**
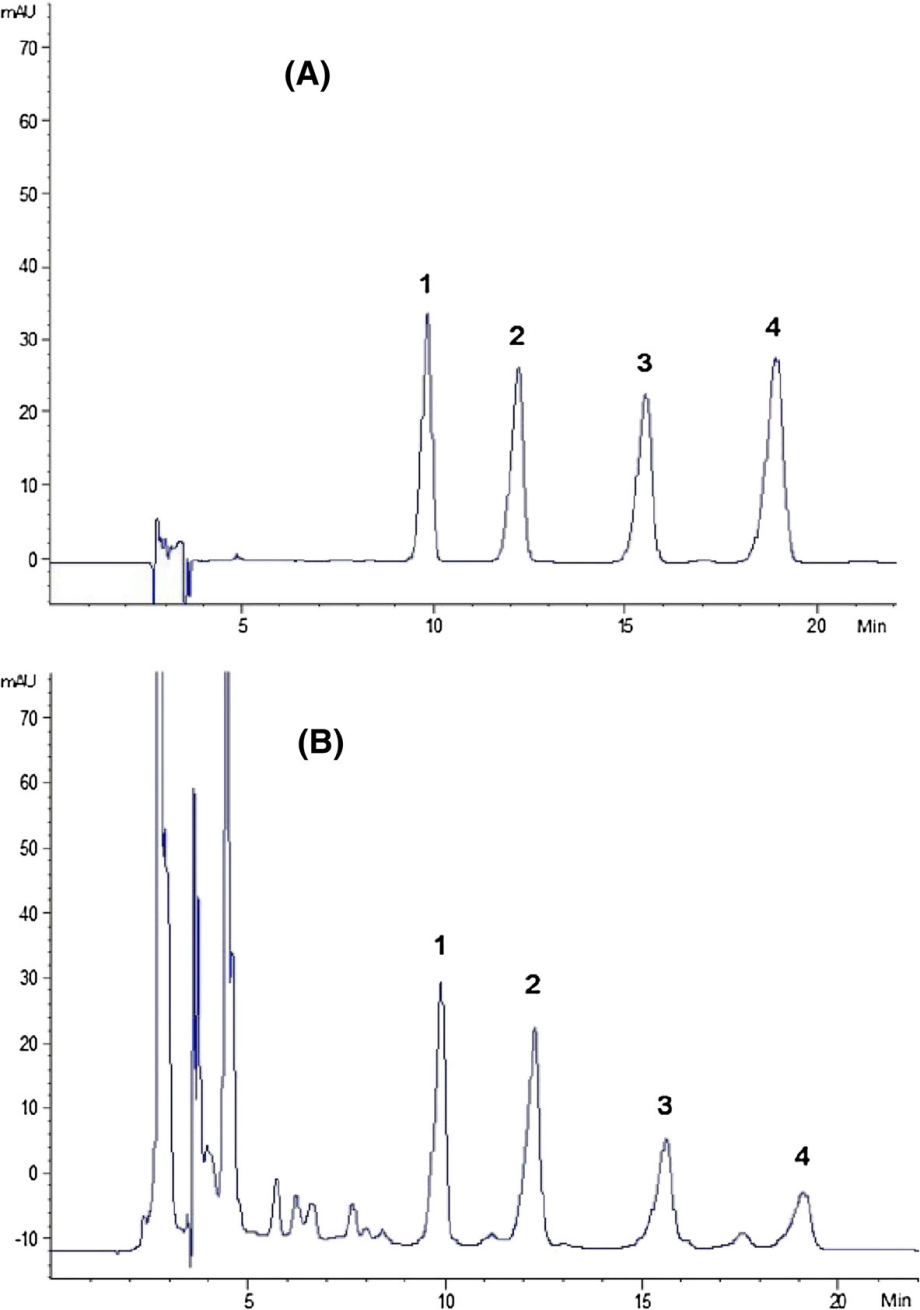
**HPLC chromatograms of *M*. *atropurpurea *leaves. (A)** Mixed reference compounds 1: rutin; 2: isoquercetin; 3: kaempferol-3-O-rutinoside; 4: astragalin. **(B)** Extract of sample MA07.

A series of mobile phases and elution programs were tested to obtain the baseline separation. All four analytes were eluted with baseline separation on the Cosmosil 5 C_18_-MS-II (4.6 × 250 mm, 5 μm) column (Shown in Figure 3).

### HPLC method validation

The method was validated by linearity, the limit of detection (LOD) and quantification (LOQ), precision, stability and accuracy.

### Calibration curves, LODs and LOQs

The calibration curves are summarized in Table 2. All calibration curves showed good linear regressions (R^2^ > 0.9992); [P < 0.001] within the testing range. The LODs and LOQs were less than 0.49 and 1.64 ng, respectively.

**Table 2:** Calibration curves, LODs, and LOQs for the four flavonoids (1–4)

**Flavonoids**	**Calibration curve**^**a**^	**R**^**2**^	**P**	**Test range (μg/mL)**	**LOD**^**b **^**(ng)**	**LOQ**^**c **^**(ng)**
**1**	y = 20.981x-29.365	0.9997	<0.001	0.98 — 500.00	0.49	1.64
**2**	y = 32.967x + 16.675	0.9995	<0.001	1.26 — 80.60	0.73	2.42
**3**	y = 22.738x + 6.6695	0.9994	<0.001	0.20 — 100.00	1.32	4.39
**4**	y = 33.411x + 10.376	0.9992	<0.001	0.20 — 100.00	1.20	3.99

^a^y = peak area and x = concentration (μg /mL).

^b^Limit of detection (S/N = 3).

^c^Limit of quantification (S/N = 10).

### Precision test

As shown in Table 3, the RSD values for overall intra-day and inter-day precision were less than 2.90 and 4.88%, for all analytes, suggesting that the developed method was precise enough for determining these flavonoids.

**Table 3:** Variations in intraday and interday precision of HPLC methods for the determination of the four flavonoids (1–4)

**Flavonoids**	**Intraday precision (n = 3)**		**Interday precision (n = 6)**	
	**Content (mg/g)**	**RSD**^**a **^**(%)**	**Content (mg/g)**	**RSD**^**a **^**(%)**
**1**	0.81 ± 0.01	1.32	0.75 ± 0.04	4.73
**2**	0.53 ± 0.02	2.90	0.52 ± 0.01	2.16
**3**	0.42 ± 0.02	2.60	0.37 ± 0.02	4.88
**4**	0.25 ± 0.01	2.83	0.23 ± 0.01	4.81

^a^RSD (%) = 100 × SD / mean.

### Stability test

The overall RSDs of peak areas at different times were less than 4.20%, indicating that the sample was stable for at least 24 h.

### Accuracy test

As shown in Table 4, the recoveries of flavonoids **1**, **2**, **3** and **4** were 97.8–100.1, 102.2–104.0, 96.1–104.6, and 97.4–104.6% (n = 3), respectively, and the RSDs were less than 5.51%, indicating that the developed method was accurate for determining these four flavonoids.

**Table 4:** Accuracy of the HPLC method for the determination of the four flavonoids (1–4)

**Flavonoids**	**Original (mg)**	**Spiked (mg)**	**Found (mg)**	**Recovery**^**a **^**(%)**	**RSD**^**b **^**(%)**
		0.13	0.27	97.8	1.99
**1**	0.14	0.15	0.29	96.0	0.38
		0.18	0.32	100.1	2.55
		0.11	0.24	102.2	2.06
**2**	0.13	0.13	0.27	103.5	3.49
		0.16	0.29	104.0	5.51
		0.07	0.16	104.6	2.56
**3**	0.08	0.09	0.17	96.1	4.55
		0.10	0.19	101.9	0.96
		0.04	0.09	97.4	3.62
**4**	0.05	0.05	0.10	104.6	0.65
		0.06	0.11	97.4	3.20

^a^Recovery (%) = 100 × (amount found - original amount) / amount spiked.

^b^RSD (%) = 100 × SD / mean.

### Quantitative analysis

The results of quantitative analysis results of eight batches of *M*. *atropurpurea* are shown in Table 5. The content of these four flavonoids were different from one batch to another, ranging from 4.31 to 0.67 mg/g, indicating the diverse quality of commercial *M*. *atropurpurea*. Raw materials of *M*. *atropurpurea* should be collected at a fixed location that enables the assurance of quality consistency, and consequently, consistency of efficacy of this herb.. The content of rutin (**1**) was highest in MA07, and lowest in MA03, while the content of isoquercetin (**2**) was highest in MA07, but lowest in MA08. The contents of kaempferol-3-*O*-rutinoside (**3**) and astragalin (**4**) were highest in MA07, but lowest in MA08.

**Table 5:** **Content of the four flavonoids (1–4) in *M*. *atropurpurea *** **leaves from different locations (mg/g) (n = 3)**

**No.**	**Location**	**Flavonoids**				
		**1**	**2**	**3**	**4**	**Total**
MA01	Chaoshan, Guangdong	0.38 ± 0.01	0.33 ± 0.00	0.18 ± 0.01	0.16 ± 0.00	1.04 ± 0.02
MA02	Qingping, Guangdong	0.51 ± 0.02	0.18 ± 0.01	0.29 ± 0.01	0.06 ± 0.00	1.04 ± 0.04
MA03	Zhanjiang, Guangdong	0.32 ± 0.03	0.34 ± 0.02	ND^a^	0.17 ± 0.01	0.83 ± 0.06
MA04	Zhixing, Guangdong	0.86 ± 0.03	0.46 ± 0.01	0.36 ± 0.01	0.13 ± 0.00	1.80 ± 0.04
MA05	Caizhilin, Guangdong	0.86 ± 0.02	0.67 ± 0.02	0.34 ± 0.00	0.28 ± 0.01	2.12 ± 0.05
MA06	Puning, Guangdong	0.72 ± 0.00	0.53 ± 0.02	0.42 ± 0.00	0.25 ± 0.00	1.92 ± 0.02
MA07	Qingping, Guangdong	1.85 ± 0.04	1.11 ± 0.02	0.95 ± 0.04	0.40 ± 0.02	4.31 ± 0.11
MA08	Zhanjiang, Guangdong	0.33 ± 0.01	0.11 ± 0.01	0.17 ± 0.01	0.06 ± 0.00	0.67 ± 0.04

^a^ND: not detected

## Conclusion

The four flavonoids in *M*. *atropurpurea* leaves could inhibit α-glucosidase activity.

## Abbreviations

1-DNJ: 1-deoxynojirimycin; N-methyl-1-DNJ: N-methyl-1-deoxynojirimycin; HPLC: High performance liquid chromatography; MWE: Mulberry leaf water extract; ESI-MS: Electric-spray ionization coupled with mass spectrometry; NMR: Nuclear magnetic resonance; pNPG: 4-nitrophenyl α-D-glucopyranoside; RM: Raw materials; RMTSR: Raw material to solvent ratio; LOD: Limit of detection; LOQ: Limit of quantification; RSD: Relative standard deviation

## Competing interests

The authors declare that they have no competing interests.

## Authors’ contributions

QWZ and WCY conceived and designed this study. HCH performed the experiments. XQZ collected the materials. HCH, SLL and QWZ wrote the manuscript. All authors read and approved the final version of the manuscript.
